# Surgical site infection in patients submitted to heart transplantation

**DOI:** 10.1590/1518-8345.0821.2700

**Published:** 2016-08-29

**Authors:** Jussara Aparecida Souza do Nascimento Rodrigues, Renata Eloah de Lucena Ferretti-Rebustini, Vanessa de Brito Poveda

**Affiliations:** 1 RN, RN, Student of the High Complexity Cardiopneumology Nurse Residency Program, Instituto do Coração, Hospital de Clínicas, Faculdade de Medicina, Universidade de São Paulo, São Paulo, SP, Brazil.; 2 PhD, Professor, Escola de Enfermagem, Universidade de São Paulo, São Paulo, SP, Brazil

**Keywords:** Perioperative Nursing, Surgical Wound Infection, Heart Transplantation, Thoracic Surgery

## Abstract

**Objectives:**

to analyze the occurrence and predisposing factors for surgical site infection in patients submitted to heart transplantation, evaluating the relationship between cases of infections and the variables related to the patient and the surgical procedure.

**Method:**

retrospective cohort study, with review of the medical records of patients older than 18 years submitted to heart transplantation. The correlation between variables was evaluated by using Fisher's exact test and Mann-Whitney-Wilcoxon test.

**Results:**

the sample consisted of 86 patients, predominantly men, with severe systemic disease, submitted to extensive preoperative hospitalizations. Signs of surgical site infection were observed in 9.3% of transplanted patients, with five (62.5%) superficial incisional, two (25%) deep and one (12.5%) case of organ/space infection. There was no statistically significant association between the variables related to the patient and the surgery.

**Conclusion:**

there was no association between the studied variables and the cases of surgical site infection, possibly due to the small number of cases of infection observed in the sample investigated.

## Introduction

Despite advances in the clinical treatment of Heart Failure (HF), in some cases heart transplantation remains as the treatment of choice^1^. Among the cardiomyopathies causing HF, Chagas disease is present in 50% of patients requiring transplantation, followed by idiopathic dilated cardiomyopathy (20.5%), ischemic cardiomyopathy (15.9%) and other etiologies, such as hypertensive cardiomyopathy, restrictive cardiomyopathy, mitral valve cardiomyopathy and peripartum cardiomyopathy (13.6%)^2^.

Heart transplantation involves, among its stages, Extracorporeal Circulation (ECC), which allows direct manipulation of the heart, still, during surgery^3^. The ECC produces systemic inflammatory response by releasing catecholamines and changing the blood fluid and the electrolyte status, allowing disorders such as edema, leukocyte clumping, low output syndrome, infections and glycemic decompensation. The higher the ECC time, the greater the physiological imbalance of the patient^4^.

In addition, patients submitted to transplantation are susceptible to various infections, with an emphasis to the Surgical Site Infections (SSI), which are the second most prevalent among the nearly two million cases of Health Care-Associated Infections (HCAI), reported annually in the United States. It should be noted that this number is likely below the actual value, because of frequent underreporting of surgical infections^5^.

The SSIs can be classified into incisional, which can be superficial or deep and organ/space and their predisposing factors are diabetes, smoking, use of systemic steroids, obesity, malnutrition and use of blood components/derivatives^6^ ^-^ ^7^.

Among the SSIs, organ/space infections such as mediastinitis and sternum osteomyelitis are present in 0.4 to 5% of cases and compromise the tissues, organs or cavities handled, which can be diagnosed from 30 to 90 days after surgical procedure. These infections are considered as severe complications; with high rates of morbidity and mortality, between 14 and 47%, representing high costs to the health system^6^ ^,^ ^8^ ^-^ ^9^.

The current scientific literature on SSIs associated with heart transplantation is limited, and the scientific literary output is focused on the incidence of SSIs in the transplanted population, that is, involving different types of transplants or, on studies on the incidence of infections in heart transplant patients, considering different sites on a single research^10^. In Brazil, only an article published in 2000, dealt specifically with SSIs among patients submitted to heart transplantation, however it only addressed mediastinitis^11^.

In view of the lack of data on SSIs in Brazil, among patients submitted to heart transplantation, the relevance of the infectious process in immunosuppressed patients and the benefits resulting from the knowledge of the factors that can prevent the onset of this infection, there was an interest in knowing the current rate of such infection in this population.

The objective of this study was to analyze the occurrence and predisposing factors for SSI among patients submitted to heart transplantation, evaluating the relationship between its occurrence and the variables related to the patient previous infection, skin preparation, duration of surgical procedure.

## Method

This is a retrospective cohort study carried out in a public university hospital of high complexity, specialized in cardiac and thoracic surgery, in the city of São Paulo. This institution serves approximately 13,000 hospitalizations per year, having performed 31 heart transplantations in adults, in 2013.

All operating rooms have exclusive circulating nurses and anesthesiologists, air conditioning, taps and antisepsis for the surgical team members activated automatically by means of a sensor. The doors are kept closed. There are temperature control and control of the number of people in the room, maximum 11.

This research was submitted to appreciation and approved by the Ethics Committee on Research on Humans (ECRH), under protocol number CAAE 30908814.7.3002.0068.

The sample was selected by convenience. Patients submitted to heart transplantation from January 2010 to January 2014 were included in the sample, totaling 176 subjects of which 90 were excluded as they were younger than 18 at the time of surgery. Thus, 86 records were analyzed from June to December 2014.

An instrument previously validated, which contained related variables was used for data collection as shown below.

- Related to the patient: age, sex, occupation, nationality, origin, hospitalization start date, date of surgery, length of stay in the Intensive Care Unit (ICU), length of stay in the infirmary and time of hospital discharge, preoperative bathing, medical diagnosis, weight, height, BMI, alcohol consumption, smoking, use of steroids, chronic diseases, ASA risk, pre-existing infections and microorganism causing the infection.

BMI was calculated by dividing body weight by height squared (kg/m²)^12^.

The ASA classification was used to evaluate the clinical status of patients, ranging from I to V. Grade I, for normal healthy patients; II, for patients with mild systemic disease; III, for patients with severe systemic disease; IV, for patients with severe systemic disease that is a constant threat to life; V, for dying patients, who are not expected to survive without the surgery; and VI, for those declared brain-dead patients, whose organs are being removed for donation purposes^13^.

- Related to the anesthetic-surgical procedure: length of preoperative hospitalization, circulatory assist device, surgical size, trichotomy, surgical technique, start time and end time of surgery and anesthesia, type of anesthesia, antibiotic prophylaxis, skin preparation, blood components/derivatives, ECC time.

According to the routine of the institution where data were collected, surgeries lasting up to two hours are considered as size I; surgeries lasting from two to four hours on average, as size II; surgeries lasting from four to six hours, as size III, and surgeries lasting more than six hours, as size IV.

- Related to the postoperative period and rehospitalization: signs and symptoms of infection, microbial culture, isolated microorganism, medical diagnosis of SSI, type of SSI, antibiotic treatment, diagnostic time, and postoperative time of rehospitalization.

Regarding the diagnostic criteria, it was used the latest international definition, which defines SSI as related to surgical procedures and classifies it according to the organic level affected: superficial incisional, deep incisional and organ/space^6^.

The surface incisional SSI occurs in the first 30 days after surgery, involving skin and subcutaneous tissues, with purulent drainage from incision or microbial growth from fluid or superficial incision tissue obtained aseptically or when the incision is opened by the surgeon, showing at least one of the signs or symptoms: pain, increased sensitivity, local edema, redness or warmth^6^.

The deep incisional SSI occurs up to 90 days after surgery and involves deep soft tissues. The diagnostic criteria are: purulent drainage from deep incision; dehiscence or wound opening by the surgeon and axillary temperature ≥38°C, pain or increased local sensitivity, abscess or other evidence of involvement of deep layers, identified during reoperation, by clinical, histo-cytopathological or imaging examination^6^.

Lastly, the organ/space SSI, which involves any organ or cavity that has been opened or manipulated during surgery. The specific criteria for this infection are purulent drainage, microbial isolation from culture or abscess or other evidence confirmed by imaging or histopathological examination^6^. A pilot test that contributed to the adjustments in the data collection instrument was carried out using the medical records of ten patients submitted to heart transplantation, whose surgery occurred after the date set for data collection, and therefore they were not included in the sample.

The statistical program SPSS, version 20.0 was used for data analysis and the results were presented in a descriptive and mathematical-statistical format, using absolute frequency and percentage. The correlation between variables was estimated by using Fisher exact test for sex, chronic diseases, ASA, smoking, previous infection, use of circulatory assist device and use of blood components/derivatives, and the Mann-Whitney-Wilcoxon test was used for age, BMI, length of preoperative hospital stay, surgery time and ECC time.

The level of significance for the analysis was set at a=5%.

## Results

The sample consisted of 86 patients aged from 19 to 69 years, average age of 42.8 years, most retired (25.6%), born in the state of São Paulo (36%), coming from the city of São Paulo (48.8 %), with ejection fraction between eight and 78%, non-drinkers (79.8%) and non-steroid users (100%).

Among the patients who had infection prior to transplantation (Table 1), Blood Stream Infection (BSI) (70.8%) and Urinary Tract Infection (UTI) (25%) are highlighted. One patient (4.2%), carrier of internal artificial ventricle, presented infection in the circuit driveline. The microorganisms most commonly found in these infections were coagulase-negative *Staphylococci*, which represented 36.4% of the sample, and it was stressed that half of these agents corresponded to *Staphylococcus epidermidis*.

**Table 1:** Distribution of the variables related to the patient and their association with signs of SSI. São Paulo, SP, Brazil, 2014

Variables		Total		Presence of SSI*		Absence of SSI*		p-value
		n	%	n	%	n	%	
Gender								1.00^†^
	Female	31	36	3	9.7	28	90.3	
	Male	55	64	5	9.1	50	90.9	
ASA^‡^								1.00^†^
	ASA^‡^ 3	2	2.3	0	0	2	100	
	ASA^‡^ 4	70	81.4	7	10.0	63	90.0	
	ASA^‡^ 5	14	16.3	1	7.1	13	92.9	
Chronic diseases								0.27^†^
	Yes	50	58.1	3	6.0	47	94.9	
	No	36	1.9	5	13.9	31	86.1	
Smoking								1.00^†^
	No	60	69.8	6	10.3	52	89.7	
	Former smoker	26	30.2	2	7.7	24	92.3	
Previous infection								0.42^†^
	Yes	25	29.8	1	4.0	24	96.0	
	No	59	70.2	7	11.9	52	88.1	

*SSI: Surgical Site Infection

†Fisher's exact test

‡ASA: American Society of Anesthesiologists

The mean BMI was 21.9 kg/m², with a standard deviation of 3.44kg/m².

Regarding the baseline diagnosis, Chagas disease was the most prevalent cardiomyopathy (38.4%), followed by idiopathic dilated cardiomyopathy (22.1%) and ischemic cardiomyopathy (17.4%). In relation to chronic diseases, 58.1% of patients had baseline diseases, highlighting the Systemic Arterial Hypertension (SAH) (19.8%), followed by Chronic Renal Failure (CRF) (18.6%).

All patients were submitted to preoperative bath using degerming chlorhexidine 2%. Subsequently, they were shaved with electric apparatus and had their skin prepared with degerming chlorhexidine 2% and alcohol 0.5%. They received balanced general anesthesia and antibiotic prophylaxis, with cefuroxime, solely, being the most used drug (58.8%). They received blood components or blood derivatives during transplantation, 82.4% (Table 2).

**Table 2:** Distribution of the variables related to the surgical anesthesia and their association with signs of SSI. São Paulo, SP, Brazil, 2014

Variables		n	Mean± Standard Deviation	Median	Minimum- maximum	p-value
Preoperative hospital stay (days)			107.4±88	84	0-553	0.23*
Surgery time (minutes)			391±130	360	235-950	0.93*
ECC time† (minutes)			125±60.1	15	63-467	0.36*
Transfused blood component						0.34^‡^
	Concentrated red blood cells	60	2.5±1.3	2	1-6	
	Fresh frozen plasma	31	3±1.6	3	1-9	
	Platelets	22	5.7±2.7	6	1-12	
	Cryoprecipitate	13	6.6±3.1	7	1-11	
	Albumin	12	2.2±0.9	2	1-4	
	Prothrombin complex	21	2±0.7	2	1-4	

*Mann-Whitney-Wilcoxon test

†ECC: Extracorporeal Circulation

‡Fisher's exact test

There was variation in surgical size, ranging from III (43.1%) to IV (56.9%). Bicaval orthotopic heart transplantation was the main technique used (95.3%).

At the time of transplantation, 39 (45.3%) patients were using circulatory assist device, and intra-aortic balloon was the most prevalent (94.9%). The ASA classification of the patients varied between three (2.3%), four (81.4%) and five (16.3%).

Among the patients submitted to transplantation, 31 (36%) died. Of these, eight (25.8%) died within 24 hours of surgery, five (16.1%) within seven days, 11 (35.5%) within 30 days and seven (22.6%) after 30 days of surgery. The main cause of death during hospitalization was graft rejection, in nine (29%) patients.

Eight patients submitted to transplantation (9.3%) showed signs of SSI in the postoperative period, and the drainage of exudate isolated from wound was the most common (37.5%). However, only four (4.7% of the total sample) received the diagnosis of SSI during hospitalization, 25.2 days after surgery on average (standard deviation-SD=15.6), minimum of 13 days and maximum of 47 days.

Among those diagnosed with SSI, 50% were classified as superficial incisional, 25% as organ/space and 25% as infection secondary to the procedure, which fulfilled all the criteria to be classified as deep incisional. The remaining four cases presented characteristics compatible with superficial incisional infection (75%) and deep incisional infections (25%).

Therefore, in the total of cases of SSI found in the sample investigated, it was observed the occurrence of five (62.5%) superficial incisional infections, two (25%) deep incisional and one (12.5%) case of organ/space infection.

The microbial culture results were available only for patients with diagnosis of SSI registered in their medical records, who showed exudate in their wounds. Presence of microorganisms was observed in three cases (37.5%): *Staphylococcus aureus* + *Klebsiella oxytoca, Candida albicans* and *Enterococcus faecium* + Coagulase-negative *Staphylococci.*

The diagnosis occurred, on average, on the twentieth day after surgery, with the patient still hospitalized. Six (7%) patients with signs of infection received antibiotic therapy with various medications (meropenem, imipenem/teicoplanin, Tazocin/vancomycin, teicoplanin/ vancomycin, meropenem/vancomycin or linezolid).

Of the total sample of patients, 53 (61.6%) were submitted to rehospitalization. The average time for rehospitalization after discharge was 90 days, with routine biopsy as the main cause in 10 (18.9%) patients, and graft rejection in five (17%) cases.

Three patients submitted to transplantation (4.9% of those hospitalized) showed signs of SSI (ventilatory dependent pain + fever, erythema and edema + incision exudate).

It is emphasized that during rehospitalization only one patient underwent microbial culture from wound exudate, which showed positive results for *Aspergillus.* Another patient who had fever during rehospitalization, had already presented pain and positive culture for *Candida albicans* in the postoperative period during his first hospitalization and was treated at that time with antibiotics and antifungals, however, the medical diagnosis of SSI occurred only during rehospitalization. The time for the diagnosis of SSI in these patients ranged from a minimum of 127 and a maximum of 342 days of postoperative.

The Fisher's exact test was used for analysis of independent variables, resulting in no significant association between sex, ASA classification, diabetes, smoking, circulatory assist device (p=1.00), chronic diseases (p=0.27), prior infection (p=0.42), use of blood components/derivatives during surgery (p=0.34) and presence of signs of SSI in the postoperative period.

The Mann-Whitney-Wilcoxon test was used to analyze the association between SSI and age (p=0.84), BMI (p=0.56), length of preoperative hospital stay (p=0.23), surgery time (p=0.93) and ECC time (p=0.36), which also showed no statistical significance for the outcome.

## Discussion

It was observed in this study that patients submitted to heart transplantation were predominantly men, with severe systemic disease, submitted to extensive preoperative hospitalization and long surgical procedures. Regarding the infection diagnosis, it was observed the presence of SSI in 9.3% of this study sample.

The rates of SSI among patients submitted to transplantation and/or cardiac surgery are variable, with evidence of only 2%^14^, similar to the values observed in this study, which found 9.4%^15^.

Gram-positive microorganisms are often involved in the etiology of SSIs, especially *Staphylococcus aureus*, coagulase-negative *Staphylococci* and *Enterococcus faecium*, although Gram-negative bacteria such as *Escherichia coli, Klebsiella oxytoca* and *Acinetobacter* may also be found. It is worth mentioning that in this study, agents commonly related to SSI were observed as described in other studies^8^ ^,^ ^15^ ^-^ ^16^, as well as other types of microorganisms, such as *Candida albicans* and *Aspergillus*, what seems to represent the peculiarity of the microbiota of the hospital where this research was carried out.

Regarding the classification of the SSIs, it is stressed the great diversity present in the medical records and registrations of infectious cases, what may affect the comparability of data. Although there are specific definitions and diagnostic criteria for each subtype of surgical site infection by topography, according to the organic level affected, it was observed in the medical records, variations in the diagnosis of infection and classifications different of those recommended by the competent bodies, such as infection secondary to the procedure. Unfortunately, this aspect is also present in other contexts and countries, since currently there are many terminologies and classifications available^17^.

It is recognized that allogeneic transfusion among patients submitted to cardiac surgery is associated with a higher risk of mediastinitis^18^. However, although 82.4% of patients analyzed in this study have received blood components or blood products, there was only one case of mediastinitis, which was not registered as such, even if it has met all diagnostic criteria.

For the diagnosis of mediastinitis, it is necessary culture of microorganisms from tissue or fluid from the mediastinum, evidence from the histopathological or anatomical examination, or at least one of the following signs or symptoms: fever (>38.0°C), chest pain or sternal instability, purulent drainage and mediastinal widening detected by imaging examination^19^.

The factors considered as predisposing to SSI are: diabetes, male gender and high BMI, however, although these variables were present in the sample investigated, none showed statistically significant association with SSI^7^ ^,^ ^15^.

One of the actions suggested for the prophylaxis of SSI is the preoperative antibiotic prophylaxis, which occurs immediately before the procedure, preferably using cephalosporins of first or second generation (cefuroxime, cefazolin). However, there is the need to individualize therapy according to previous infection/colonization of the donor or recipient^7^ ^,^ ^20^.

In the analysis, 58.8% of patients received only cefuroxime as prophylaxis during surgery, whereas the others received a variety of other drugs (meropenem, tazocin, vancomycin, ceftriaxone, clindamycin, ciprofloxacin), solely or combined.

The implementation by the hospital, of prophylaxis protocols with antibiotics in a systematic fashion, prevents the occurrence of SSI, reducing the microbial load present at the time of surgery. It is worth highlighting, however, that the implementation of the use of these drugs should be based on scientific evidence, ensuring success and reducing the risks of increasing bacterial resistance, through microorganisms selection^21^ ^-^ ^22^.

Amongst the 86 patients submitted to the surgical procedure and included in this study, 31 (36%) died. Similar frequency (38.9%) was found in a study at national level on cardiac surgery^15^, however, when only deaths directly related to mediastinitis were analyzed, this rate dropped to 20.4%^2^. It should be stressed that the major cause of death in this study was associated with graft loss, occurring mostly in periods longer than seven days. This may be associated with clinical complications arising from the severity of the patients or due to infectious complications such as SSI, since one of the patients who died as result of septic shock, presented SSI.

Deep SSI is a postoperative complication that increases the in-hospital mortality in patients submitted to heart transplantation^23^. Moreover, most of infectious conditions, even those in other sites, including pneumonia and bloodstream infections, are diagnosed in the postoperative period, which reinforces the importance of post-discharge surveillance^24^.

In this regard, it is emphasized that three cases were submitted to rehospitalization due to SSI, strengthening the importance of post-discharge surveillance to achieve reliable estimates on the cases of SSI in healthcare institutions. It is emphasized that the lack of standardization of the criteria for SSI screening, in the period after discharge, can hinder the detection of SSI cases^17^.

A study carried out with 3,663 patients submitted to general surgery showed rates of SSI of 10%. Of these, 48% were diagnosed after hospital discharge and 15.6% were submitted to rehospitalization. Most rehospitalizations occurred in the first week after discharge^25^.

The same study indicates that the surveillance after hospital discharge must be intense for all surgical patients between the first and the second week of hospital discharge, period with an increased chance of infections detection. Only a directed telephone call would be sufficient for this investigation, which could help in the reduction of hospital costs^25^, since, in general, patients with serious infections, such as deep and organ/space infections are often rehospitalized, increasing the institutional expenses.

Finally, the prevention and control of cases of SSI involve the multifactorial and multidisciplinary approach, highlighting the role of nurses who must work intensively in all phases of the surgical experience, in order to minimize these complications, by means of the implementation of preventive measures, approach of the risk factors and active search of the potential cases of infection.

## Conclusion

It can be concluded that the sample consisted predominantly of men, with severe systemic disease, submitted to preoperative hospitalization and extensive surgical procedures, with 9.3% of transplant recipients showing signs of surgical site infection and drainage of the wound exudate was the most prevalent sign (37.5%).

There was no statistically significant association between the variables related to the patient or between variables related to the surgery with cases of SSI. Perhaps this aspect can be explained by the small number of cases of infection detected in the sample.

In this way, it is suggested to conduct new studies preferably prospective in which it will be possible to evaluate the cases of SSI within 30 days, in a larger sample. This method of collecting data will allow the application of standardized measures for evaluation of cases and control of other variables not available in the medical records.

## References

[1] Lindenfeld J, Albert NM, Boehmer JP, Collins SP, Ezekowitz JA, Givertz MM (2010). Executive summary HFSA 2010 comprehensive heart failure practice
guideline. J Card Fail.

[2] Silva EA, Carvalho DV (2012). Transplante cardíaco complicações apresentadas por pacientes durante a
internação. Esc Anna Nery.

[3] Dienstmann C, Caregnato RCA (2013). Circulação extracorpórea em cirurgia cardíaca um campo de trabalho
para o enfermeiro. Rev SOBECC.

[4] Torrati FG, Dantas RAS (2012). Circulação extracorpórea e complicações no período pós-operatório
imediato de cirurgias cardíacas. Acta Paul Enferm.

[5] Magill SS, Edwards JR, Bamberg W, Beldavs ZG, Dumyati G, Kainer MA (2014). Multistate point-prevalence survey of health care-associated
infections. N Engl J Med.

[6] Center for Disease Control (2015). CDC/NHSN - Surgical Site Infection (SSI) Event.

[7] Mangram AJ, Horan TC, Pearson ML, Silver LC, Jarvis WR, the Hospital infection control practices advisory committee (1999). Guideline for the prevention of surgical site infection,
1999. Infect Control Hosp Epidemiol.

[8] Deniz H, Gokaslan G, Arslanoglu Y, Ozcaliskan O, Guzel G, Yasim A (2012). Treatment outcomes of postoperative mediastinitis in cardiac surgery;
negative pressure wound therapy versus conventional treatment. J Cardiothor Surg.

[9] Tiveron MG, Fiorelli AI, Mota EM, Mejia OAV, Brandão CMA, Dallan LAO (2012). Fatores de risco pré-operatórios para mediastinite após cirurgia
cardíaca análise de 2768 pacientes. Rev Bras Cir Cardiovasc.

[10] Dorschner P, McElroy LM, Ison MG (2014). Nosocomial infections within the first month of solid organ
transplantation. Transpl Infect Dis.

[11] Stolf NAG, Fiorelli AI, Bacal F, Camargo LF, Bocchi EA, Freitas A (2000). Mediastinitis after Cardiac Transplantation. Arq Bras Cardiol.

[12] Graham R, Mancher M, Wolman DM, Greenfield S, Steinberg E (2011). Clinical Practice Guidelines We Can Trust..

[13] American Society of Anesthesiologists (ASA) (2015). ASA Physical Status Classification System.

[14] Ho JK, Sung-Ho J, Jae JK, Joon BK, Suk JC, Tae-Jin Y (2013). Early postoperative complications after heart transplantation in adult
recipients Asian medical center experience. Korean J Thorac Cardiovasc Surg.

[15] Silva QCG, Barbosa MH. (2012). Fatores de risco para infecção de sítio cirúrgico em cirurgia
cardíaca. Acta Paul Enferm.

[16] Shinagawa N, Taniguchi M, Hirata K, Furuhata T, Fukuhara K, Mizugucwi T (2014). Bacteria isolated from surgical infections and its susceptibilities to
antimicrobial agents--special references to bacteria isolated between April 2010
and March 2011. Jpn J Antibiot.

[17] Leaper D, Tanner J, Kiernan M (2013). Surveillance of surgical site infection more accurate definitions and
intensive recording needed. J Hosp Infect.

[18] Ang LB, Veloria EN, Evanina EY, Smaldone A (2012). Mediastinitis and blood transfusion in cardiac surgery a systematic
review. Heart Lung.

[19] Center for Disease Control (2015). CDC/NHSN - Surveillance Definitions for Specific Types of Infections.

[20] Bacal F, Souza JD, Fiorelli AI, Mejia J, Marcondes-Braga FG, Mangini S (2010). II Diretriz brasileira de transplante cardíaco. Arq Bras Cardiol.

[21] Finkelstein R, Rabino G, Mashiach T, Bar-El Y, Adler Z, Kertzman V (2014). Effect of preoperative antibiotic prophylaxis on surgical site
infections complicating cardiac surgery. Infect Control Hosp Epidemiol.

[22] Najjar PA, Smink DS (2015). Prophylactic antibiotics and prevention of surgical site
infections. Surg Clin North Am.

[23] Dorschner P, McElroy LM, Ison MG (2014). Nosocomial infections within the first month of solid organ
transplantation. Transplant Infect Dis.

[24] Gelijns AC, Moskowitz AJ, Acker MA, Argenziano M, Geller NL, Puskas JD (2014). Cardiothoracic surgical trials network (CTSN). J Am Coll Cardiol.

[25] Gibson A, Tevis S, Kennedy G (2014). Readmission after delayed diagnosis of surgical site infection a focus
on prevention using the American College of Surgeons national surgical quality
improvement program. Am J Surg.

